# QA-kNN: Indoor Localization Based on Quartile Analysis and the kNN Classifier for Wireless Networks

**DOI:** 10.3390/s20174714

**Published:** 2020-08-21

**Authors:** David Ferreira, Richard Souza, Celso Carvalho

**Affiliations:** 1 Department of Electronics and Computing Engineering (DTEC)—Electrical Engineering Graduate Program (PPGEE), Federal University of Amazonas, Manaus, AM 69067-005, Brazil; ferreirad08@gmail.com; 2 Department of Electrical and Electronics Engineering, Federal University of Santa Catarina, Florianópolis, SC 88040-900, Brazil; richard.demo@ufsc.br

**Keywords:** indoor localization, quartile analysis, kNN classifier, wireless networks

## Abstract

Considering the variation of the received signal strength indicator (RSSI) in wireless networks, the objective of this study is to investigate and propose a method of indoor localization in order to improve the accuracy of localization that is compromised by RSSI variation. For this, quartile analysis is used for data pre-processing and the k-nearest neighbors (kNN) classifier is used for localization. In addition to the tests in a real environment, simulations were performed, varying many parameters related to the proposed method and the environment. In the real environment with reference points of 1.284 density per unit area (RPs/m^2^), the method presents zero-mean error in the localization in test points (TPs) coinciding with the RPs. In the simulated environment with a density of 0.327 RPs/m^2^, a mean error of 0.490 m for the localization of random TPs was achieved. These results are important contributions and allow us to conclude that the method is promising for locating objects in indoor environments.

## 1. Introduction

The concept of Internet of Things (IoT) proposed to turn previously traditional devices into intelligent devices, which can make decisions when performing tasks and which are able to inform parameters of their functioning. IoT is an important paradigm in the globalization of technology, where up to 40 billion connected devices are expected in the future [1]. Therefore, indoor localization is a topic of great interest in the scope of IoT, since it can contribute to the improvement of applications in several areas, such as smart homes, health, and industry, among others [2]. Although the global positioning system (GPS) provides acceptable data of physical localization in an outdoor environment, its precision and usefulness are compromised as the dimensions of the environment decrease, mainly, in an indoor environment [3].

The indoor localization is the target of several works. These proposals can be based on geometric techniques such as trilateration [4], which use radius/distances estimated by time of arrival (ToA), time difference of arrival (TDoA) or angle of arrival (AoA) to form circles and identify an intersection point. Alternatively, it can be based on techniques that use specific pattern/features such as the fingerprint [5], which associate measurements previously collected from the received signal strength indicator (RSSI) with a new measurement to identify a corresponding position. Unlike ToA, TDoA and AoA, RSSI is provided by hardware drivers from different manufacturers from different technologies such as RFID (Radio Frequency IDentification), Zigbee, Wi-Fi and FM (Frequency Modulation) radio, among others [6,7].

In indoor environments with multiple obstacles, traditional systems have difficulties in locating objects with high precision, due to the problems of signal interference and variation of raw measurements [8,9]. However, the selection of the most appropriate techniques allows the implementation of localization systems with high accuracy and reliability, which can assist in applications and services in different areas. These applications include, among others, marketing, as it assists in displaying advertisements and offers based on the user’s localization; tracking products (e.g., merchandise, hospital equipment and large components, etc.); and, mainly, medical assistance, which makes it possible to locate doctors and patients for better care.

New approaches are being proposed to reduce the estimation errors of these systems, for example, the use of methods based on machine learning algorithms for the localization of users and moving objects [10,11,12]. Recent research is dedicated for determining techniques for building suitable datasets for machine learning algorithms, such as normalizing and representing raw data, identifying, and removing redundant data, as well as mathematical models for obtaining the data. However, most contributions group RSSI measurements in a generalized way and do not consider behavioral aspects, such as the degree of spread of data present in sets affected by different obstacles [13,14,15,16].

The omission of these aspects can lead to an increase in the error of localization estimates. In this sense, this article proposes a method for indoor location using statistical characteristics in the pre-processing of RSSI measurements. To this end, preliminary experiments were carried out with the aim of corroborating the extraction of discriminating characteristics. The use of machine learning is considered to propose the localization method.

To evaluate the proposed method, experiments were conducted in a real environment with an area of 3.50 m × 3.56 m. In these practical experiments, Wi-Fi networks were used, but other technologies can be used in the proposed method, such as RFID, ZigBee, FM Radio and UWB (Ultra-Wide-Band), among others. Subsequently, the method was evaluated in simulated environments with areas of 7 m × 7 m, 14 m × 14 m, 28 m × 28 m and 56 m × 56 m. In contrast to the methods tested without the variation of RSSI, the simulator was developed based on measurements of RSSI in the real environment, considering path loss and shadowing, in order to make a more adequate and close modeling of a real environment.

The main contribution of this work is the significant improvement of the localization through a promising method and fully integrable with the systems already proposed in the literature. In addition, computational modeling, and simulation to evaluate the proposed method in larger environments allow the performance of exhaustive tests at random TPs (Test Points).

## 2. Related Works

The work of Kuntal and Karmakar [17] proposes a localization algorithm based on the k-nearest neighbors (kNN) classifier for IEEE (Institute of Electrical and Electronics Engineers) 802.11 networks. The localization of the moving object is obtained by the centroid, that is, by the weighted average of the coordinates of the k nearest neighbors. The MATLAB software [18] was used in the simulations, the log-normal shadowing model to obtain the RSSI and the Euclidean distance to verify similarity. To obtain a better performance, different parameters were varied. Among them, the effect of the number of access points (APs), the value of k and the shadowing factor (*X*_σ_) in the calculation of the RSSI was examined, as this factor changes when a change occurs in the environment.

The results indicate that the use of seven APs reflects a better performance, since the error decreased as the number of APs increased, however the error became constant when the number of APs was greater than seven. The value of k = 4 also obtained better performance in the simulations, presenting a localization error of 0.8 m. The value of the shadowing factor was initially defined with σ = 0 and the errors increased as σ increased.

The methodology used by [17] may not present satisfactory results on a real test platform, since the representation of RSSI in the offline phase using the log-normal shadowing model with σ = 0 does not present conformity with the RSSI obtained through the occurrence of disturbances in the online phase, limiting itself only to simulated tests.

The work of Torres-Sospedra et al. [15] presents a comprehensive study using 51 metrics of distance and similarity, 4 alternatives to represent the raw data and a threshold based on the RSSI values. The tests were performed using the public database UJIIndoorLoc [19]. The article indicates that kNN-based localization systems are improved only by selecting the appropriate configuration.

In view of the possible configurations presented, the best result was achieved using the similarity function Sørensen [20] combined with the data representation “powed”, which was proposed by the authors to address the logarithmic nature of raw data from RSSI, and k = 13. This k value was obtained after tests with odd values from 1 to 23, with a 95.2% hit rate and a 6.19 m localization error. The application of a proposed threshold method to remove Wi-Fi signals with very low intensity did not show significant improvements, but decreased the performance of the localization system when the intensity values close to the average values were discarded and, as a conclusion, the method was not suggested.

Just as the removal of low levels of intensity impairs the performance of the system, the use of the “powed” representation may not present satisfactory results in the classification algorithm. This is because discriminating variables or attributes are decisive for a classification algorithm, and the normalized values of low levels of intensity by the representation “powed” have high similarity.

The work developed by Jedari et al. [21] investigated the performance of three classifiers based on machine learning, including kNN, the JRip (Java Repeated Incremental Pruning) classifier proposed in [22] and the random forest algorithm to estimate indoor localization. Measurements were made using 50 reference points (RPs). For each RP, 30 raw measurements from the RSSI of 86 Aps were obtained.

With a hit rate of 77.40%, the kNN classifier proved to be superior to JRip, however, it did not present a better performance when compared to random forest (with a hit rate of 91%) due to the limitations of the chi-squared technique (*χ*^2^) that was applied to remove redundant data before they were used by the kNN algorithm.

According to McHugh [23], limitations of *χ*^2^ include requirements regarding the size of the dataset, since a large amount of data interpretation errors is presented when using a large number (20 or more) of classes or attributes.

The work by Salamah et al. [16] used principal component analysis (PCA) to improve the performance of indoor localization systems. The PCA identifies the redundancy between multiple attributes, and it transforms the dataset into a set with new attributes or main components ordered by the importance that each has in terms of information, being able to eliminate attributes with less importance and represent the dataset with a reduced dimension [24].

The performance of the proposed method was tested using the kNN, decision tree, random forest, and support vector machine (SVM) classifiers. The experiments were conducted in a real environment in 45 RPs using smartphones to collect the RSSI from 6 APs. The results show that the kNN with k = 2 and based on the first three components obtained the best performance in the dynamic experiments, presenting a localization mean error of 1.71 m and 3 m with an accuracy of 60% and 79%, respectively.

In the method addressed by [16], the localization was calculated by the centroid of the RPs, as well as in [17]. This calculation presents the possibility of locating objects in positions close to the RPs and where RSSI measurements were not collected.

In the work of Tang et al. [25], to improve the localization accuracy that is affected by the RSSI variation, the following were proposed: a RSSI–probability transformation algorithm for the construction of a new dataset formed by RSSI values and their corresponding probabilities; and the grouping of RPs in clusters, calculating the weight of each AP to represent small regions in the localization environment.

To verify the performance of the proposal, a localization system called LocNeedle was developed based on the kNN classifier, adopting the Bhattacharyya distance [26] as a metric similarity measure. This system was compared with the RADAR system proposed by [4] and LocNeedle using datasets only with the average RSSI of each AP. The best experimental result was achieved using the LocNeedle system combined with the RSSI–probability transformation algorithm, with a localization error of 4.5 m.

The probability of a given RSSI value belonging to a position is a promising alternative to circumventing the problem with highly similar RSSI values, being more efficient than the removal of the low RSSI values adopted by [15]. This demonstrates that the use of new representations of the RSSI alone may not be enough to improve the accuracy; however, additional attributes with specific information, which characterize the behavior of the RSSI, guarantee a better influence on the results of localization.

The works of [27,28] use low-cost UWB devices in their proposals for internal location for NLOS (non-line-of-sight) environments. In particular, the work of [27] uses machine learning (ML) techniques to analyze various RSSI measurement scenarios and identify measurements that showed variations related to NLOS propagation characteristics of the signals. The work of [28] proposes and analyzes the performance of a location system that combines location algorithms with ML techniques applied to a previous classification process to reduce problems related to the propagation of signals in indoor environments.

However, we note that proposals [27,28] do not consider the transformation of RSSI samples using quartiles, as we proposed in our article. We add as a comment that the differential of our proposal is to use data transformation using quartiles in order to create signatures that show statistical differences between the RSSI samples collected in different RPs/TPs. This approach allows us to more accurately identify the positioning of a WSTA (Wireless Station) to be located.

The works presented in this section used machine learning algorithms with datasets generated by RSSI, and the vast majority obtained the best results using the kNN algorithm in their proposals bt using different configurations to obtain a satisfactory result.

Among the various configurations used by the related works, there is the method of representing the RSSI samples collected in each RP or TP. The work [17] uses raw RSSI values to characterize the signature of a given RP. The problem with this approach is that the same or similar raw values can be obtained in different RPs and come from different APs, making it impossible to identify each RP/TP in a unique way. The work of [15] creates representations for the RSSI samples called “exponential” and “powed” by using exponentiation and potentiation operations associated with normalization of the raw RSSI values read in RPs/TPs. This approach has a similar problem to [17], since equal/similar values of RSSI can be obtained from readings performed in different RPs and APs, not characterizing RPs in a unique way. The work of [21] uses chi-squared representation (*χ*^2^) to select, for each RP, the RSSI readings that have a greater degree of independence; however, the work obtains a better low accuracy value equal to 56% of correct location. The work of [16] uses the average value of the RSSI samples collected in each RP to represent the RSSI readings performed in each RP/TP. However, different sample sets collected from two different RPs, for example, can result in the same average value, not uniquely identifying each RP/TP. Finally, the work of [25] uses the probability of reading a RSSI value in each RP to create fingerprints. However, different RPs with the same or different distances from a certain reference AP can have similar probability of occurrence of the RSSI readings, which can cause identification/localization errors.

Unlike all the proposals mentioned, we performed, in each RP/TP, readings of RSSI values from each AP in the environment and divided the set of samples into quartiles to represent the RSSI readings from each AP. With the use of quartiles (Q1, Q2 and Q3) we have a larger set of characteristics that can uniquely represent a particular RP/TP. For example, for a RP1, if the set of RSSI readings from an AP1 result in the representation of quartiles Q1 = A, Q2 = B, and Q3 = C and, for a RP2 the representation in quartiles is still similar with values Q1 = A, Q2 = B and Q3 = D, we will still have quartile Q3 to differentiate RPs/TPs 1 and 2. With this approach, as previously mentioned and as will be presented in the following sections, for a real environment with a density of 1284 RPs/m^2^, we obtained a localization mean error (*ME*) equal to zero and, in a simulated environment with a density of 0.327 RPs/m^2^, we obtained a *ME* of 0.490 m for the situation where the TPs were random.

## 3. Material and Methods

### 3.1. Experimental Scenario

**Figure 1:**
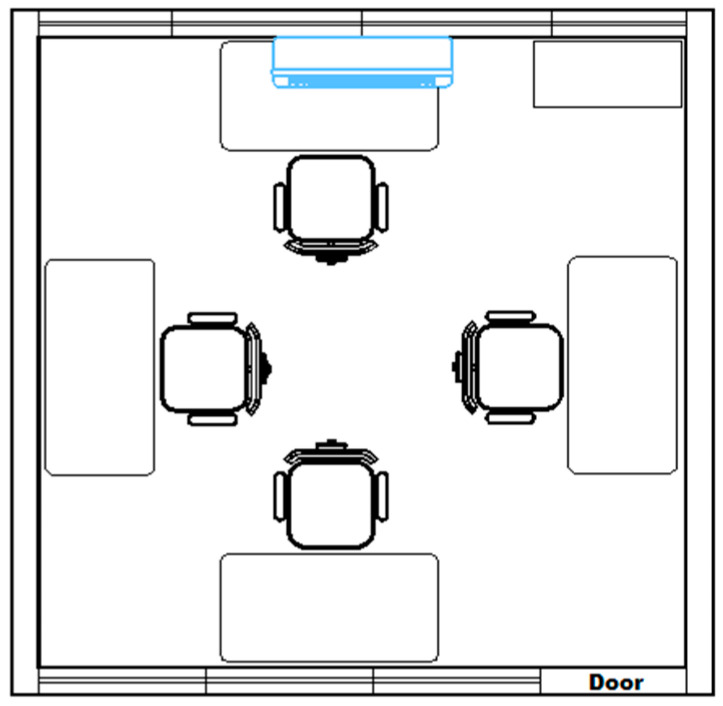
Experimental scenario layout.

Experiments were conducted using the RSSI to estimate the localization of a moving object in any position within the indoor environment. Figure 1 shows the layout of the experimental scenario, which has dimensions of 3.50 m × 3.56 m, contains a cabinet (illustrated in the upper right corner of the figure), and four rectangular tables in the center of each wall. 

**Figure 2:**
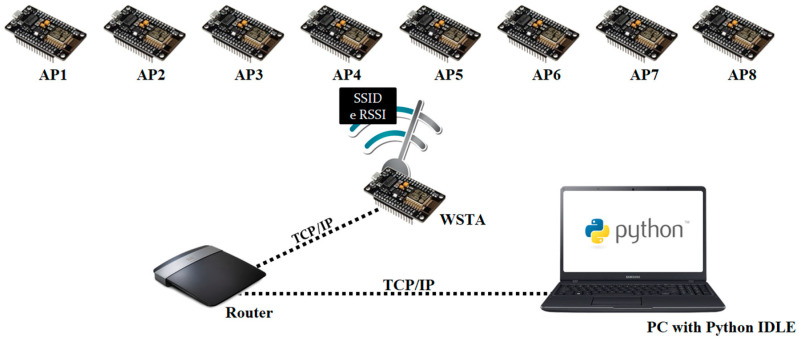
Connection diagram of the elements that make up the localization system.

In the localization system, NodeMCU modules, powered by external 4.5 to 9 V batteries, were used, and they consist of an ESP8266 microcontroller, a UART–USB (Universal Asynchronous Receiver Transmitter- Universal Serial Bus) interface and a 3.3 V voltage regulator [29]. NodeMCU can be used in station mode (STA), access point (AP) or both (STA + AP) and can be configured in 802.11 b/g/n standards. In this work, the modules were pre-configured in the standard 802.11 g, which has a transmission power of 17 dBm and a minimum sensitivity of −98 dBm for the data rate of 1 Mbps [30]. According to [31], the ESP 8266 module has a communication range of around 100 m. Figure 2 illustrates the connection diagram of the elements that make up the system, where eight modules in AP mode are illustrated.

We used a software Wi-Fi Analyzer to observe that channels 1, 6 and 11 were the most occupied by APs installed close to the environment, possibly because there was no overlap between these channels. In view of this, channel 4 for the APs of the localization system was selected to reduce Wi-Fi interference. The mobile node to be located was configured in STA mode and for convenience it was called WSTA (wireless station). No directional antenna was attached to the modules.

In general, the WSTA collects information about the 8 APs, such as the SSID (service set identifier) and RSSI and, stores it on an embedded HTTP (Hyper Text Transfer Protocol) server. When requested, the data are sent to the personal computer (PC) with Python IDLE (Python’s Integrated Development and Learning Environment) using the router. Finally, the PC processes the data to obtain the localization of the WSTA.

**Figure 3:**
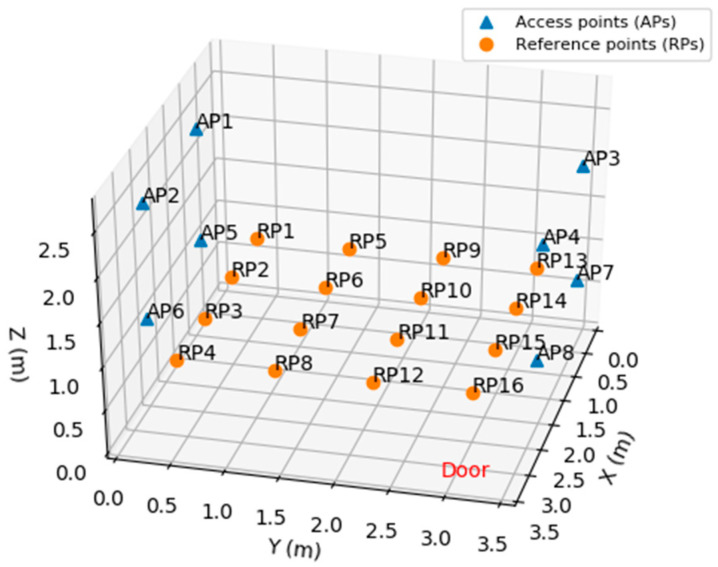
Positioning of access points (APs) and reference points (RPs).

For the experiments, a division of the indoor environment was adopted in 16 zones with RPs positioned in the center of each zone and at a height of 0.87 m. The RPs were used as collection points for the RSSI measurements from the APs. Figure 3 illustrates the positioning of the APs and RPs, where the 8 APs are represented by triangles and the 16 RPs are represented by circles. The indoor environment has dimensions of 3.50 m length (X), 3.56 m width (Y) and 2.80 m height (Z), with a density of 1.284 RPs/m^2^.

Table 1 shows the set of coordinates of the APs and RPs in the indoor environment where the experiments were carried out.

### 3.2. Relationship between the Distance and RSSI

In order to analyze the relationship between the transmitter–receiver separation distance (AP-WSTA) and the RSSI, preliminary experiments were performed on adjacent RPs 1 and 2. Variations and the behavior of power loss in the propagation of electromagnetic signals were observed. Two techniques presented in the literature have been tested to express, in indoor environments, the RSSI behavior as a function of distance: the quadratic approximation technique used in [32,33] and the log-normal shadowing technique, also called log-normal propagation loss, used in [17,34,35].

**Figure 4:**
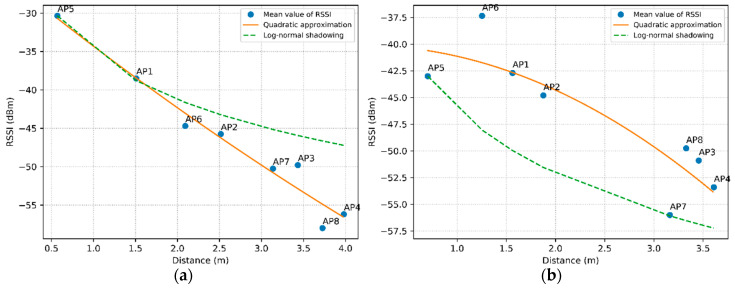
Comparison between log-normal shadowing, quadratic approximation and the average to obtain the received signal intensity indicator. (**a**) Reference point 1 and (**b**) reference point 2.

Figure 4 shows the RSSI curves as a function of distance in practical experiments. The blue circles correspond to the arithmetic mean of 20 RSSI measurements of all the APs in RPs 1 and 2, respectively, in Figure 4a,b. Estimating the average RSSI from 20 readings is sufficient to reduce the squared error of the variations [36]. The solid orange curve corresponds to the quadratic approximation technique and the green dashed curve corresponds to the log-normal shadowing technique.

Figure 4a shows the results of the experiments carried out in RP1. RSSI decreases exponentially with increasing distance in the techniques of quadratic approximation and log-normal shadowing, as expected; however, the average RSSI of the measurements collected showed some variations. Figure 4b shows the RSSI results obtained in RP2. As the average RSSI presents greater variations for close distances, not having a strict relation in the inverse proportion of the square of the distance, the techniques of quadratic approximation and log-normal shadowing tend to be further away from the average value of RSSI in this scenario of experimentation.

The different variations in RSSI measurements are due to the absence of line of sight (LOS) as a result of the lack of directional antennas, and to the propagation phenomena of electromagnetic waves, such as path loss, shadowing and multipaths. Thus, the theoretical techniques used in some works without the addition of the RSSI variation cannot express the loss of signal power in practical tests, as they are limited to simulated experiments. Four observations were possible in the experiments:

1. Signals collected in a RP with the same distance in relation to two APs had different mean RSSI values;
2. Signals collected in a RP with different distances in relation to two APs had the same average RSSI values;
3. Signals collected from two RPs with the same distance in relation to an AP had different mean RSSI values;
4. Signals collected from two RPs with different distances from an AP had the same average RSSI value.

In this experiment, it was found that the average RSSI remained similar in tests conducted on different days. Thus, methods related to the average RSSI can provide more information about the RSSI measurements in each RP. In this way, in this paper we used methods based on specific RSSI patterns to determine the localization of the WSTA, as can be seen in the following sections.

### 3.3. Data Representation Using Quartile Analysis

In the evaluation of the preliminary experiments, the relevance of conducting studies for the extraction of more discriminating characteristics among the APs is noted. Thus, a possible solution found in this work was the calculation of quartiles from the RSSI measurements of multiple APs.

Quartile analysis is a statistical method used to assess the central trend and data dispersion. Quartiles partition a partially ordered set (poset) into four equal parts. The first quartile (*Q*_1/4_) or lower quartile, delimits the 25% of the lowest observations; the second quartile (*Q*_2/4_), or median, separates the 50% smallest from the 50% largest observations; and the third quartile (*Q*_3/4_), or upper quartile, delimits the 25% largest observations [37].

Several definitions are found in the literature for calculating quartiles [38]. Thus, a generalized equation for the computational/statistical calculation of quartiles is defined in this article. From a poset (*S*, ≤) with *n* elements, the quartile value *Q_p_* is estimated through linear regression between the elements *x*_⌊*i*__⌋_ and *x*_⌊*i*__⌋_ + 1, where the position ⌊*i*⌋ is determined as a function of the respective percentage *p*, according to the equation:

(1) $$Q_{p}=x_{\left\lfloor i\right\rfloor }+\left(x_{\left\lfloor i\right\rfloor +1}-x_{\left\lfloor i\right\rfloor }\right)\times \left(i-\left\lfloor i\right\rfloor \right)\;with\;i=\left(n-1\right)\times p+1,$$

where ⌊*i*⌋ is the integer part of *i*.

For highly asymmetric data that are affected by outliers, *Q*_2/4_ is more efficient than the average and does not require a previous exploratory analysis. In addition, the interquartile range (*IQR* = *Q*_3/4-_ *Q*_1/4_) is a relatively robust statistical measure compared to the standard deviation [39].

**Figure 5:**
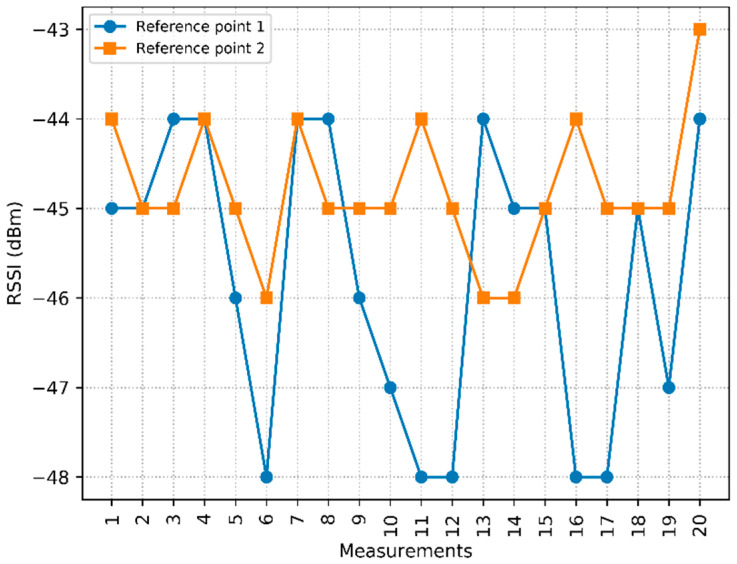
RSSI measurements from access point 2 (AP2) collected at RP1 and RP2.

Figure 5 shows the raw RSSI measurements from AP2 collected in the adjacent RPs 1 and 2, whose distances to AP2 are 2.52 m and 1.88 m, respectively.

Table 2 shows the quartile values of AP2 analyzed in RPs 1 and 2. Although collected at different distances, the measurements result in the same *Q*_2/4_, that is, the same RSSI of −45 dBm. Therefore, the need to use the features *Q*_1/4_ and/or *Q*_3/4_ to differentiate between two different RPs is understood.

In Table 2, the difference in *Q*_1/4_ values can be seen, due to the greater variation in measurements collected in RP1, and due to the influence of obstacles, which cause the lowest RSSI readings to be further away from *Q*_2/4_. This behavior can be seen in the blue solid curve of Figure 5, where measurements are observed in RP1 with lower RSSI values when compared to the solid orange curve, referring to RP2. Such behavior was also observed in new measurements. To make the data suitable for classification algorithms, such as the kNN algorithm that was implemented in related works, the simultaneous use of the three quartiles as discriminating features is evaluated.

### 3.4. Localization Method

To estimate the localization, the proposed method employs the k-nearest neighbors (kNN) classification algorithm. kNN is one of the simplest and most effective classification algorithms in supervised learning [40]. It is non-parametric, that is, it does not need the data to have a specific distribution (e.g., gaussian or exponential), and it has as a hyperparameter the number of nearest neighbors (k). In general, large number of neighbors can reduce or increase the performance of the algorithm, which is subject to the extraction of discriminating features to represent the data.

In training, the classification model is created by a set of previously classified instances and the k value is defined empirically for better precision. In the classification, a test instance (new instance) is introduced in the trained classifier. Traditionally, the classifier examines the classes of the k training instances most similar (nearest) to the test instance based on distance metrics, such as Manhattan, Euclidean or Minkowski. Subsequently, it attributes the test instance to the majority class, that is, the class most represented by the k training instances [41].

In this localization method, the kNN classification algorithm adopts the Euclidean distance as a function of similarity and the quartile analysis in the data representation. The method is evaluated using two prediction approaches: coordinates of the majority RP and coordinates of the centroid of the RPs. 

#### 3.4.1. Coordinates of the Majority Reference Point

In this approach, the WSTA coordinates are estimated in the coordinates of the majority RP, according to the equation:

(2) $$f_{i}=\max\{f_{1},\ldots ,f_{n}\}\to ({\hat{x}}_{WSTA},{\hat{y}}_{WSTA},{\hat{z}}_{WSTA})=(x_{i},y_{i},z_{i}),$$

that is, if the frequency of occurrence *f_i_* is the highest observed frequency, then the ($\hat{x}$_WSTA_, $\hat{y}$_WSTA_, $\hat{z}$_WSTA_) coordinates are equal to the (*x_i_*, *y_i_*, *z_i_*) coordinates of the RP*i*. When there is a tie in the frequencies of multiple RPs, the coordinates of the RP associated with the nearest neighbor are chosen to the estimated localization.

The coordinates of the majority RP are an approach commonly used when the kNN algorithm is indicated for the localization problem, as in the works done by [15,21,25]. However, this makes it possible to estimate localization only in the positions where the RSSI measurements were collected.

#### 3.4.2. Coordinates of the Centroid of the Reference Points

In this approach, the WSTA coordinates are estimated using the centroid, that is, the average of the coordinates of the RPs associated with the k nearest neighbors, weighted by the respective frequencies of the occurrences of each RP (*f_i_*) according to the equation:

(3) $$({\hat{x}}_{WSTA},{\hat{y}}_{WSTA},{\hat{z}}_{WSTA})=\left(\frac{\sum_{i=1}^{k}f_{i}x_{i}}{\sum_{i=1}^{k}f_{i}},\frac{\sum_{i=1}^{k}f_{i}y_{i}}{\sum_{i=1}^{k}f_{i}},\frac{\sum_{i=1}^{k}f_{i}z_{i}}{\sum_{i=1}^{k}f_{i}}\right),$$

where ($\hat{x}$_*WSTA*_, $\hat{y}$_*WSTA*_, $\hat{z}$_*WSTA*_) are the coordinates of the centroid and *f_i_* is the weight for the RP *i*.

The coordinates of the centroid were proposed by [16,17] and make possible to estimate localization in any position within the indoor environment, which includes positions where no RSSI measurements were collected.

## 4. Experiments

**Figure 6:**
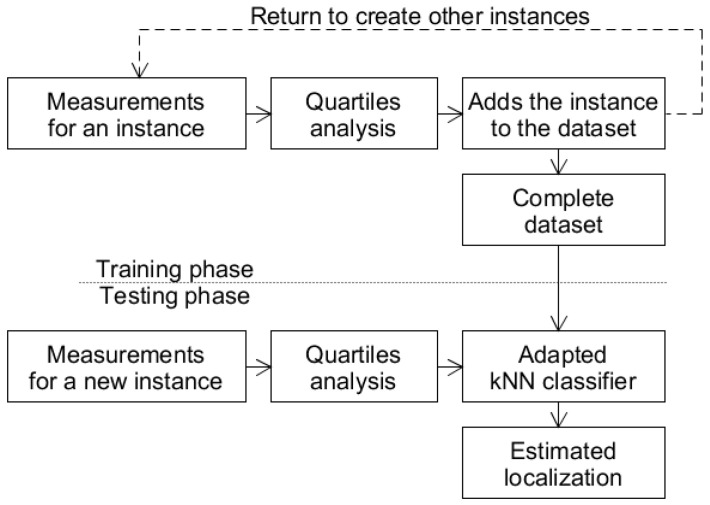
Flow diagram common to methods I and II.

In the experiments, the two approaches were evaluated: the majority reference point (RP) among the k nearest neighbors described in Section 3.4.1 and the centroid of the RPs of the k nearest neighbors described in Section 3.4.2. These approaches are denoted in this section as method I and method II, respectively. Figure 6 presents the training and test flow diagram common to both methods.

As shown in Figure 6, methods I and II involve two phases. In the training phase, the WSTA collects raw RSSI measurements sent by the APs periodically and records these data in known positions in the physical space (called RPs). Then, according to the quartile analysis, discriminant characteristics are extracted to establish a dataset suitable for classification. New instances are created until the dataset has been balanced with the same number of instances per RPs. In the test phase, the system also records the RSSI values received from the APs, but in unknown positions. Then, according to the kNN algorithm adapted to each tested method, the actual localization of the WSTA is estimated.

### 4.1. Dataset

To create a previously classified instance, the WSTA is manually positioned in the RP corresponding to the class, where raw RSSI measurements of installed APs are collected. These measurements are organized in the matrix

(4) $$A_{m\times n}=\left[\begin{array}{ccccc} RSSI_{11} & RSSI_{12} & RSSI_{13} & \cdots & RSSI_{1n}\\ RSSI_{21} & RSSI_{22} & RSSI_{23} & \cdots & RSSI_{2n}\\ \vdots & \vdots & \vdots & \ddots & \vdots \\ RSSI_{m1} & RSSI_{m2} & RSSI_{m3} & \cdots & RSSI_{mn} \end{array}\right],$$

where each column contains *m* measurements from a given AP. The quartiles (*Q*_1/4_, *Q*_2/4_ and *Q*_3/4_) are then calculated for the APs based on their respective measurements. Thus, the instance is represented by a vector *X* of 3*n* elements/features:

(5) $$X=\begin{array}{cccc} \overset{AP_{1}}{\overbrace{[\begin{array}{ccc} Q_{1/4} & Q_{2/4} & Q_{3/4} \end{array}}} & \overset{AP_{2}}{\overbrace{\begin{array}{ccc} Q_{1/4} & Q_{2/4} & Q_{3/4} \end{array}}} & \cdots & \overset{AP_{n}}{\overbrace{\begin{array}{ccc} Q_{1/4} & Q_{2/4} & Q_{3/4} \end{array}]}} \end{array}.$$

To create instances previously classified into multiple classes, the WSTA is positioned sequentially in all RPs and the above steps are repeated until all instances are created. In the experiments, 50% of the instances were used for training and 50% for tests to prevent any influence (bias) on the results. To obtain a balanced training set, that is, with the same number of instances between classes, a stratified random sampling was performed. All measurement data considered in this work are publicly available in [42].

### 4.2. Tests

In order to obtain the lowest localization error and a low computational cost, the classification process of the test set instances was subjected to 49 quantitative treatments defined by combinations of varying numbers of APs (n) and the hyperparameter k of the classification algorithm, as shown in Table 3.

Table 3 shows that the number of tested APs starting with a value of n = 2, as the use of only one AP would result in low accuracy since in this case the probability of RSSI repetition would be high. Additionally, the hyperparameter k was tested with odd values from 1 to 13. These treatments allow us to measure the best number of APs and the appropriate number of nearest neighbors, that is, the best pair (n, k). When the best precision pair (n, k) is defined with the lowest possible values, there is less hardware consumption and a reduced processing time, since less data need to be collected and processed by the algorithm.

After the tests, the performances of the two methods were compared with the 3-PCA (Principal Component Analysis) method presented in a practical way in the work conducted by [16] and the Powed–Sørensen (PS) method presented in a practical way by [15]. For comparison, the 3-PCA and PS methods were reproduced and physically tested in the same environment as the methods covered in this work due to the differences in the test environments used by the authors, such as the size, the number of RPs and the arrangement of the furniture in the environment.

As both methods, 3-PCA and PS, estimate the localization in the coordinates of the majority RP, method I was compared to the PS method, which uses the Sørensen similarity function combined with the “powed” data representation. Furthermore, because both estimate the localization using the centroid of the RPs, method II was compared with the 3-PCA method, which combines the Euclidean distance similarity function with the PCA analysis data representation. The methods covered in this work and the methods presented in the literature and implemented for comparison purposes are presented in Table 4.

## 5. Results and Discussion

### 5.1. Localization Mean Error

To evaluate the performance of the implemented methods, the localization mean error (*ME*) was used. The *ME* in meters (m) is defined as:

(6) $$ME=\frac{1}{N}\sum_{i=1}^{N}\sqrt{{\left(x_{i}-{\hat{x}}_{i}\right)}^{2}+{\left(y_{i}-{\hat{y}}_{i}\right)}^{2}},$$

where (*x_i_*, *y_i_*) are the actual coordinates, (${\hat{x}}_{i},\;{\hat{y}}_{i}$) are the estimated WSTA coordinates for each test and N is the number of corresponding tests.5.1.1. Cumulative Distribution Function

First, to analyze the performance of all four methods in all tests, the cumulative distribution function (CDF) graph of the *ME* was constructed.

**Figure 7:**
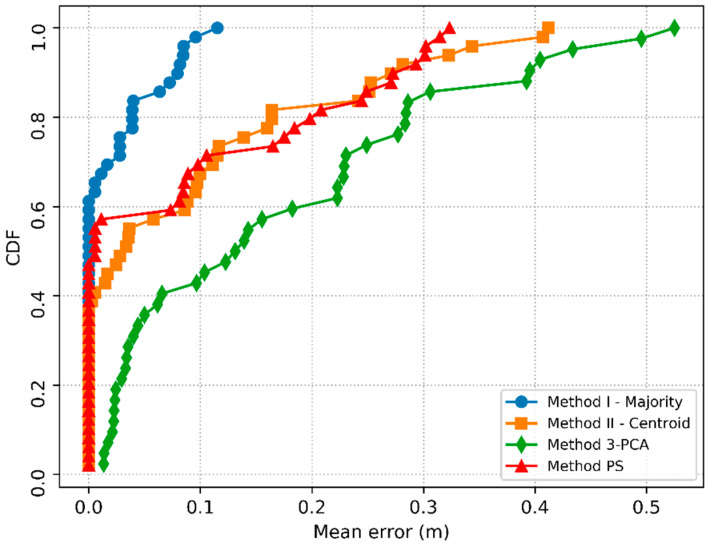
Cumulative distribution function (CDF) for localization errors.

Figure 7 shows the CDF of the localization errors for the four implemented methods. Method I produced the best results, where all tests (100%) showed an error of less than 0.12 m, whereas methods II, 3-PCA and PS showed an error of less than 0.12 m in only 73.47, 45.24 and 71.43% of the tests, respectively. It was also observed that the PS method reached a maximum error lower than that of methods II and 3-PCA, however, the maximum error of PS (0.323 m) was almost three times greater than the maximum error of method I (0.115 m).

Still in Figure 7, it is interesting to note that methods II and 3-PCA had the worst performances; this result is possibly due to both methods estimating the localization through the centroid. In this case, the localization *ME* can be improved by reducing the distance between RPs, using more RPs in the environment. However, the use of more RPs involves more manual efforts to create the dataset [43].

#### 5.1.1. Variation in the Number of APs and *K* Hyperparameter

**Figure 8:**
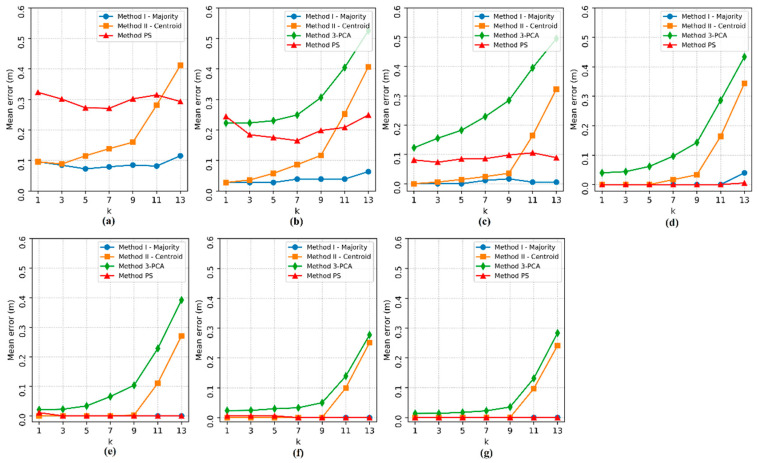
Localization mean error varying the number of access points (n) and the hyperparameter k. The subfigures show the tests for each number of access points: (**a**) 2 APs, (**b**) 3 APs, (**c**) 4 APs, (**d**) 5 APs, (**e**) 6 APs, (**f**) 7 APs and (**g**) 8 APs.

Then, the effect of the number of APs (n) and the influence of the number of nearest neighbors (k) on the localization estimates of the four implemented methods were analyzed. The 3-PCA method was tested only from three APs, as the transformation used in this method requires at least three attributes in the dataset. The localization *ME* for each pair (n, k) can be seen in Figure 8.

In Figure 8, it is possible to see that the *ME* decreases significantly as the number of APs increases for methods I and PS, but when the number of APs is n ≥ 6, the *ME* becomes constant for method I for any k value, while the localization *ME* for the PS method could still be slightly improved with n = 8. Method II and 3-PCA, which use the weighted average of the coordinates of the RPs, also saw a reduction in the *ME* as the number of APs increased, however, the values assumed by k had significant influence, because as the hyperparameter k increases, the errors tend to increase.

Regarding to the lowest localization *ME* obtained and the best pair (n, k) that define each method, both proposed methods I (n = 4, k = 1) and II (n = 4, k = 1) obtained null error, as seen in Figure 8c; PS (n = 5, k = 1) also obtained null error, as seen in Figure 8d; and 3-PCA (n = 8, k = 1) showed a *ME* of 0.013 m, as seen in Figure 8g. Methods I, II and PS also showed zero *ME* with other pairs (n, k), however, the aforementioned pairs have the lowest possible values, implying less hardware processing usage and a reduced processing time since less data need to be processed.

Note that the proposed methods I and II are equivalent with k = 1 since the average of the coordinates of a single RP is equal to the coordinates of this RP.

#### 5.1.2. Collection Time and Processing Time

In addition, the average data collection and processing times were calculated considering the best pair (n, k) for each one method. The results are shown in Table 5.

With the performed tests, one can note that the best time is achieved by method I (n = 4, k = 1), presenting 605.493 milliseconds (ms). Note that 98.8% of this time involves the WSTA scanning and collection of RSSI samples from the four APs. Therefore, the proposed method I (n = 4, k = 1) is the one with the smallest average total time and smallest average processing time.

## 6. Simulated Experimentation

### 6.1. Estimation of Simulation Parameters (Propagation Loss Exponent and Shadowing)

To evaluate the performance of methods I and II (majority and centroid) in environments of larger dimensions and in random positions, an algorithm for simulating the effects of radio frequency signal propagation in wireless networks was developed using the log-normal shadowing model, defined as:

(7) $$L(d)=L(d_{0})+10\alpha \log\left(\frac{d}{d_{0}}\right)+X_{\sigma },$$

where *L* is the path loss, α is the path loss exponent that represents how this loss increases with respect to the distance, *d*_0_ is the reference distance that is determined by measurements near the transmitter, *d* is the transmitter–receiver separation distance (T–R), and *X*_σ_ is a random variable with zero mean and standard deviation σ [44].

**Figure 9:**
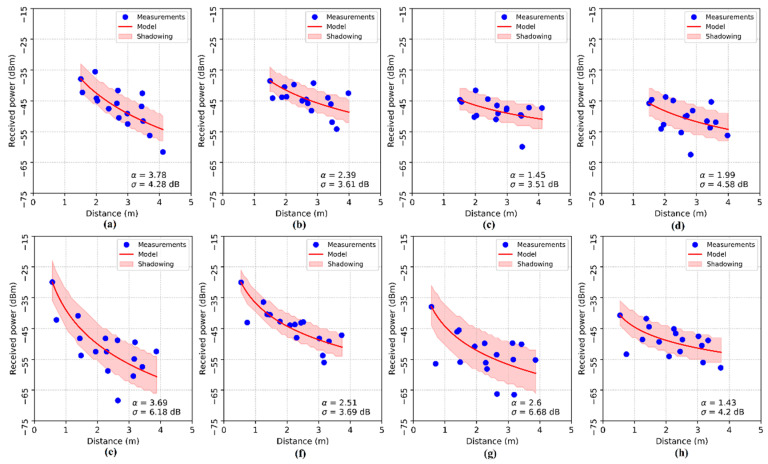
Log-normal shadowing model. The subfigures show the estimated parameters for each access point: (**a**) AP 1, (**b**) AP 2, (**c**) AP 3, (**d**) AP 4, (**e**) AP 5, (**f**) AP 6, (**g**) AP 7 and (**h**) AP 8.

The α and σ values for each AP (transmitter) were obtained from the RSSI readings collected in each RP of the real environment setup of Figure 3. The results can be seen in Figure 9.

From the resulting model, power could be estimated over greater distances by the equation:

(8) $$P_{r}(d)=P_{r}(d_{0})-10\alpha \log\left(\frac{d}{d_{0}}\right),$$

where *P_r_*(*d*) + *X*_σ_ is the power estimated at the distance of interest *d*, and *P_r_*(*d*_0_) is the power received at the reference distance.

### 6.2. Practical versus Simulated

To validate the simulator, experiments were carried out in a simulated environment with the same dimensions as the real environment (3.50 m × 3.56 m). Thus, 8 APs and 16 RPs were simulated with the same coordinates shown in Table 1 for the real environment, with a density of 1.284 RPs/m^2^. In the localization tests, 16 test points (TPs) were tested with the same positions as the RPs.

**Figure 10:**
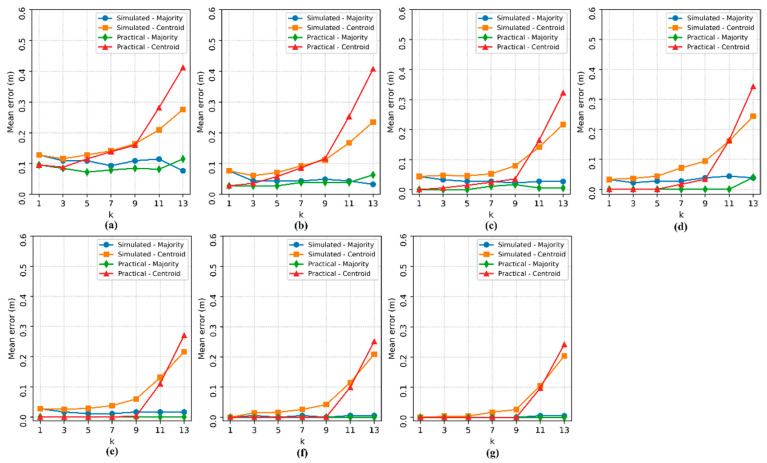
Localization mean error in the simulated environment and in the real environment. The subfigures show the tests for each number of access points: (**a**) 2 APs, (**b**) 3 APs, (**c**) 4 APs, (**d**) 5 APs, (**e**) 6 APs, (**f**) 7 APs and (**g**) 8 APs.

As in the real environment experiments, the number of APs (n) tested varied between 2 and 8, and the hyperparameter k of the kNN classifier was tested with odd values between 1 and 13. Then, a comparison was made between the performance results in the experiments in a simulated environment and in the experiments in a real environment based on the mean errors, which are shown in Figure 10.

The results observed in Figure 10 show that the errors decreased as n increased. In both experiments, real and simulated, there was no significant influence of the hyperparameter k on the method based on the majority RP. However, the errors of the method based on the centroid of the RP increased as k increased. 

The correlation between the localization errors in the simulated environment and in the real environment is remarkable, with the comparison of the experiments according to the variables n and k. From the experimental results, the simulator is presented as a possible reinforcement for localization tests in larger environments.

### 6.3. Expanded Simulation

From the computer simulation, it is possible to test the effect of the density of RPs per unit area (RPs/m^2^) in indoor environments. For a constant number of RPs, the density of RPs/m^2^ decreases as the area of the environment increases. In addition, the use of the simulator allows us to measure the performance of exhaustive localization tests in random TPs, exemplifying cases closer to reality.

To assess the performance of the proposed methods in larger dimensions environments, simulations with areas of 7 m × 7 m, 14 m × 14 m, 28 m × 28 m and 56 m × 56 m were considered, respectively. In all experiments, 8 APs equally distributed over the surface of two opposite walls, 16 RPs evenly distributed, and 16 TPs randomly distributed in each area were simulated. The APs and RPs were simulated at the same heights shown in Table 1 and the TPs were simulated at 0.87 m in height. 

**Figure 11:**
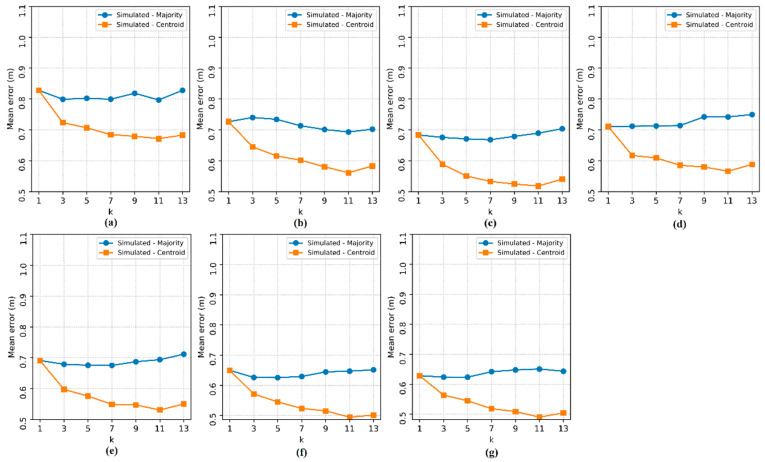
Localization mean error in the simulated environment with an area of 7 m × 7 m. The subfigures show the tests for each number of access points: (**a**) 2 APs, (**b**) 3 APs, (**c**) 4 APs, (**d**) 5 APs, (**e**) 6 APs, (**f**) 7 APs and (**g**) 8 APs.

**Figure 12:**
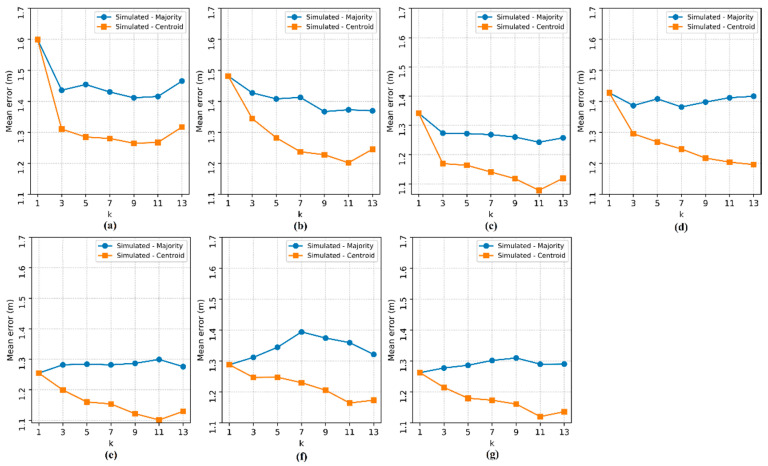
Localization mean error in the simulated environment with an area of 14 m × 14 m. The subfigures show the tests for each number of access points: (**a**) 2 APs, (**b**) 3 APs, (**c**) 4 APs, (**d**) 5 APs, (**e**) 6 APs, (**f**) 7 APs and (**g**) 8 APs.

**Figure 13:**
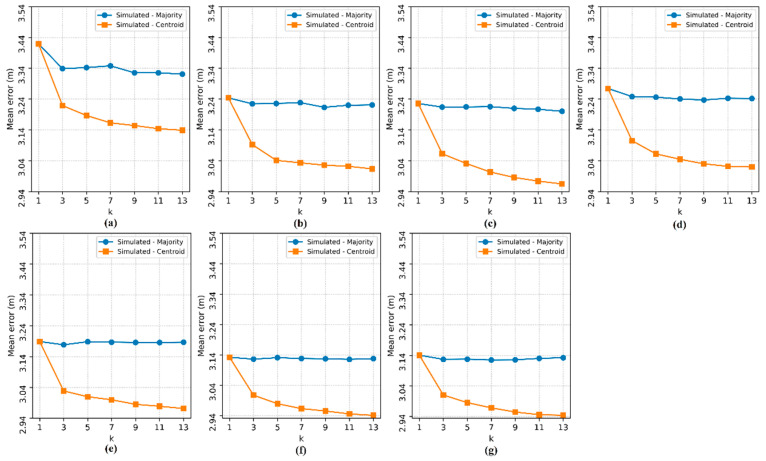
Localization mean error in the simulated environment with an area of 28 m × 28 m. The subfigures show the tests for each number of access points: (**a**) 2 APs, (**b**) 3 APs, (**c**) 4 APs, (**d**) 5 APs, (**e**) 6 APs, (**f**) 7 APs and (**g**) 8 APs.

**Figure 14:**
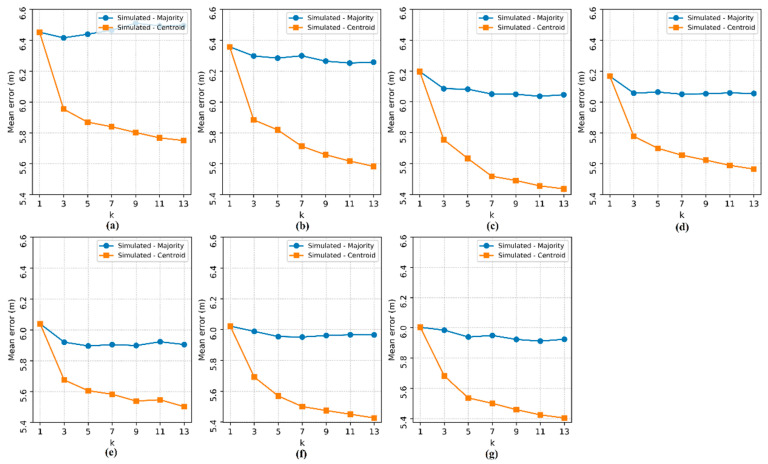
Localization mean error in the simulated environment with an area of 56 m × 56 m. The subfigures show the tests for each number of access points: (**a**) 2 APs, (**b**) 3 APs, (**c**) 4 APs, (**d**) 5 APs, (**e**) 6 APs, (**f**) 7 APs and (**g**) 8 APs.

Figure 11, Figure 12, Figure 13 and Figure 14 show the performance curves of the proposed methods obtained by simulating larger environments.

In Figure 11, Figure 12, Figure 13 and Figure 14, it is possible to observe that regardless of the dimensions considered, the reduction in the localization *ME* is still a trend ensured by the increase in the number of APs, presenting insignificant variations in the rise of the localization *ME*. It is also noted that, in general, there is no significant influence of the hyperparameter k on the method based on the majority RP. However, the errors of the method based on the centroid of the RPs decreased as k increased. This is only due to the random distribution of TPs.

**Figure 15:**
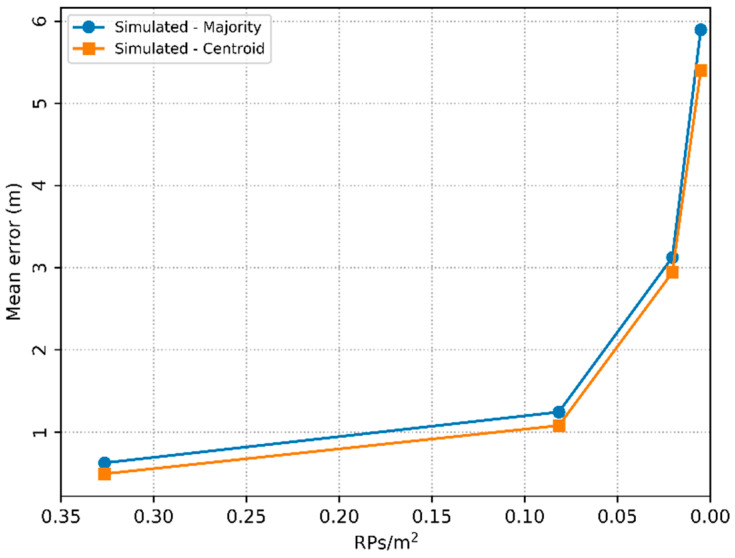
Localization mean error in the function of the density of RPs per unit area.

The results for k = 1 obtained by the two methods are equivalent, but for k = 3, the method based on the centroid of the RPs is better than that based on the majority RP. These results attest, therefore, that the calculation of the centroid allows the estimation in any position in the area, including the random positions of the TPs. Considering the increase in the environment area and, consequently, the reduction in the density of RPs/m^2^, the errors of both methods increased relatively, as shown in Figure 15.

The data presented in the graph of Figure 15 are the smallest errors of both methods, obtained empirically with areas of 7 m × 7 m, 14 m × 14 m, 28 m × 28 m and 56 m × 56 m. In all experiments, 16 RPs evenly distributed in each area were simulated, resulting in densities close to 0.327, 0.082, 0.020, 0.005 RPs/m^2^, respectively. The number of APs (n) and the hyperparameter k form an adjusted pair and are represented by (n, k). Thus, the method based on the majority RP obtained mean errors of 0.624, 1.242, 3.125 and 5.896 m with the respective pairs (8, 5), (4, 11), (8, 7) and (6, 5), and the method based on the centroid of the RPs obtained mean errors of 0.490, 1.078, 2.941 and 5.540 m with the respective pairs (8, 11), (4, 11), (7, 13) and (8, 13).

In view of the above, in order to maintain a considerable RPs/m^2^ density and subsequently minimize the localization *ME*, the number of RPs must be increased proportionally with the area of the indoor environment. 

## 7. Conclusions

In this work, a localization method was investigated using quartile analysis in the representation of the data to improve the localization accuracy, which is compromised by the problems of signal interference and variation of the raw RSSI measurements, caused mainly by obstacles. To estimate the localization of the moving object, the proposed method employs the kNN classification algorithm with two prediction approaches: coordinates of the majority RP and coordinates of the centroid of the RPs.

Experiments were carried out in real and simulated environments to evaluate the performance of the proposal, varying the number of APs (n), the hyperparameter k and the density of RPs per unit area (RPs/m^2^) in order to validate a localization method of high performance. To verify the viability of the proposed method, the proposal was compared with two localization methods present in the literature in practical experiments. The results show that the performance of the proposed method surpasses the indoor localization of the methods reported in the literature. During the tests, in the situation of better performance of the proposed method, a localization *ME* of zero was obtained with the use of only four APs and with k = 1 in an environment with a density of 1.284 RPs/m^2^. In view of the results in a simulated environment, the use the centroid of RPs for a robust localization in any position is proposed.

The results with k = 1 ensure that the quartile analysis adequately represents the RSSI values, since the instances acquire discriminating features. Based on these observations, the possibility of accurately locating in indoor environments with problems of variation of RSSI in wireless networks is verified, which validates the objective of this work. 

As future work, we highlight the development of a self-adaptation mechanism for the use of previously existing APs as a means to prevent unwanted disconnection of the APs from interfering with the localization results. Temporary shutdown can result in increased localization time due to the delay imposed by data inconsistency, caused by the lack of acquisition of RSSI data from APs during the shutdown period. Additionally, it is recommended to use techniques for selecting a single quartile with the more discriminant value to represent the dataset with a reduced size. The reduction in the dataset allows the implementation of the localization system in devices with little storage space in an embedded manner.

## Figures and Tables

**Table 1:** Coordinates of access points (APs) and reference points (RPs).

Identifier	X Coordinate	Y Coordinate	Z Coordinate
Access Points			
AP1	0.78	0.00	2.27
AP2	2.48	0.00	2.27
AP3	0.78	3.56	2.27
AP4	2.48	3.56	2.27
AP5	0.78	0.00	0.97
AP6	2.48	0.00	0.97
AP7	0.78	3.56	0.97
AP8	2.48	3.56	0.97
Reference Points			
RP1	0.4375	0.445	0.87
RP2	1.3125	0.445	0.87
RP3	2.1875	0.445	0.87
RP4	3.0625	0.445	0.87
RP5	0.4375	1.335	0.87
RP6	1.3125	1.335	0.87
RP7	2.1875	1.335	0.87
RP8	3.0625	1.335	0.87
RP9	0.4375	2.225	0.87
RP10	1.3125	2.225	0.87
RP11	2.1875	2.225	0.87
RP12	3.0625	2.225	0.87
RP13	0.4375	3.115	0.87
RP14	1.3125	3.115	0.87
RP15	2.1875	3.115	0.87
RP16	3.0625	3.115	0.87

**Table 2:** Quartiles of the AP2 with RSSI measured in the RP1 and RP2.

	AP2		
	*Q*_1/4_ (dBm)	*Q*_2/4_ (dBm)	*Q*_3/4_ (dBm)
**RP1**	−47.25	−45	−44
**RP2**	−45	−45	−44

**Table 3:** Initial, final values and increments of the measured variables.

Parameter	Initial Value	Final Value	Increment
n	2	8	1
k	1	13	2

**Table 4:** Techniques of the covered methods and the methods presented in the literature.

Method		Similarity Function	Data Representation	Obtaining the Coordinates
**Proposals**	I	Euclidian distance	Quartiles analysis	Majority RP
	II	Euclidian distance	Quartiles analysis	Centroid of RPs
**Literature**	3-PCA	Euclidian distance	PCA	Centroid of RPs
	PS	Sørensen	Powed	Majority RP

**Table 5:** Collection time and processing time.

Method	Collection Time (ms)	Processing Time (ms)	Total (ms)
I (n = 4, k = 1)	598.205	7.288	605.493
II (n = 4, k = 1)	598.205	8.390	606.595
3-PCA (n = 8, k = 1)	1196.411	8.333	1204.744
PS (n = 5, k = 1)	747.757	8.027	755.784
